# Remote monitoring technologies in Alzheimer’s disease: design of the RADAR-AD study

**DOI:** 10.1186/s13195-021-00825-4

**Published:** 2021-04-23

**Authors:** Marijn Muurling, Casper de Boer, Rouba Kozak, Dorota Religa, Ivan Koychev, Herman Verheij, Vera J. M. Nies, Alexander Duyndam, Meemansa Sood, Holger Fröhlich, Kristin Hannesdottir, Gul Erdemli, Federica Lucivero, Claire Lancaster, Chris Hinds, Thanos G. Stravopoulos, Spiros Nikolopoulos, Ioannis Kompatsiaris, Nikolay V. Manyakov, Andrew P. Owens, Vaibhav A. Narayan, Dag Aarsland, Pieter Jelle Visser, the RADAR-AD Consortium, Maximilian Buegler, Richard Fischer, Robbert Harms, Irene B. Meier, Ioannis Tarnanas, Ana Diaz, Jean Georges, Dianne Gove, Casper de Boer, Marijn Muurling, Pieter Jelle Visser, Ioannis Kompatsiaris, Ioulietta Lazarou, Lampros Mpaltadoros, Spiros Nikolopoulos, Asterios Papastergiou, Thanos Stavropoulos, Dimitris Strantsalis, Holger Froehlich, Martin Hoffman-Apitius, Meemansa Sood, Nikolay Manyakov, Vaibhav A. Narayan, Jerry G. Novak, Dorota Religa, Emilia Schwertner, Juraj Secnik, Bengt Winblad, Dag Aarsland, Pauline Conde, Amos Folarin, Grace Lavelle, Andrew P. Owens, Andrew McCarthy, Aidan Nickerson, Janneke Boere, Bruna Consiglio, Yoanna Daskalova, Alexander Duyndam, Irene Kanter-Schlifke, Vera J. M. Nies, Pieter Stolk, Herman Verheij, Neva Coello, Jelena Curcic, Gul Erdemli, Tilo Hache, Kristin Hannesdottir, Alex Sverdlov, Vanessa Vallejo, Eric Yang, Ariel Dowling, Rouba Kozak, Melissa Naylor, Rodrigo Palma dos Reis, Gene Shin, Joris Borgdorff, Elisa Cirillo, Keyvan Hedayati, Nivethika Mahasivam, Aidan Doherty, Chris Hinds, Ivan Koychev, Claire Lancaster, Sebastien Libert, Federica Lucivero, Yuhao Wu, Andre Durudas

**Affiliations:** 1 Alzheimer Center Amsterdam, Department of Neurology, Amsterdam Neuroscience, Vrije Universiteit Amsterdam, Amsterdam UMC, Amsterdam, the Netherlands; 2 Takeda Pharmaceuticals International Co., Cambridge, MA USA; 3 Department of Neurobiology, Care Sciences and Society, Karolinska Insitutet, Stockholm, Sweden; 4 Department of Psychiatry, University of Oxford, Oxford, UK; 5 Lygature, Utrecht, The Netherlands; 6 Fraunhofer Institute for Algorithms and Scientific Computing, University of Bonn, Bonn, Germany; 7 Novartis Institutes for BioMedical Research, Cambridge, MA USA; 8 Ethox and Welcome Centre for Ethics and Humanities, University of Oxford, Oxford, UK; 9 Big Data Institute, University of Oxford, Oxford, UK; 10 Information Technologies Institute, Center for Research and Technology Hellas (CERTH-ITI), Thessaloniki, Greece; 11 Data Science and Clinical Insights, Janssen Research & Development, Beerse, Belgium; 12 Department of Old Age Psychiatry, Institute of Psychiatry, Psychology & Neuroscience, King’s College London, London, UK; 13 Janssen Neuroscience Research & Development, Titusville, NJ USA; 14 Centre for Age-Related Medicine, Stavanger University Hospital, Stavanger, Norway; 15 Department of Psychiatry and Neuropsychology, School for Mental Health and Neuroscience, Maastricht University, Maastricht, the Netherlands; 16 https://www.radar-ad.org/

**Keywords:** Alzheimer’s disease, Remote monitoring technologies, Wearable technologies

## Abstract

**Background:**

Functional decline in Alzheimer’s disease (AD) is typically measured using single-time point subjective rating scales, which rely on direct observation or (caregiver) recall. Remote monitoring technologies (RMTs), such as smartphone applications, wearables, and home-based sensors, can change these periodic subjective assessments to more frequent, or even continuous, objective monitoring. The aim of the RADAR-AD study is to assess the accuracy and validity of RMTs in measuring functional decline in a real-world environment across preclinical-to-moderate stages of AD compared to standard clinical rating scales.

**Methods:**

This study includes three tiers. For the main study, we will include participants (*n* = 220) with preclinical AD, prodromal AD, mild-to-moderate AD, and healthy controls, classified by MMSE and CDR score, from clinical sites equally distributed over 13 European countries. Participants will undergo extensive neuropsychological testing and physical examination. The RMT assessments, performed over an 8-week period, include walk tests, financial management tasks, an augmented reality game, two activity trackers, and two smartphone applications installed on the participants’ phone. In the first sub-study, fixed sensors will be installed in the homes of a representative sub-sample of 40 participants. In the second sub-study, 10 participants will stay in a smart home for 1 week.

The primary outcome of this study is the difference in functional domain profiles assessed using RMTs between the four study groups. The four participant groups will be compared for each RMT outcome measure separately. Each RMT outcome will be compared to a standard clinical test which measures the same functional or cognitive domain. Finally, multivariate prediction models will be developed. Data collection and privacy are important aspects of the project, which will be managed using the RADAR-base data platform running on specifically designed biomedical research computing infrastructure.

**Results:**

First results are expected to be disseminated in 2022.

**Conclusion:**

Our study is well placed to evaluate the clinical utility of RMT assessments. Leveraging modern-day technology may deliver new and improved methods for accurately monitoring functional decline in all stages of AD. It is greatly anticipated that these methods could lead to objective and real-life functional endpoints with increased sensitivity to pharmacological agent signal detection.

## Background

Alzheimer’s disease (AD) is a neurodegenerative disease characterized by amyloid β (Aβ) and tau pathology [1]. Symptoms associated with the disease include extensive cognitive and functional decline, resulting in patients gradually losing their ability to carry out activities of daily life (ADL) and function independently. In the early stage of the disease, people with demonstrably high levels of Aβ and tau pathology present little or no cognitive and functional symptoms (prodromal/preclinical AD) that are detectable with the available tools to date. As the disease progresses, cognition and function decline towards the syndromic stages of mild cognitive impairment (MCI) and dementia [2], with the extent of functional impairment being the key feature differentiating between these stages. The conventional method for measuring such decline is through questionnaires, patient and caregiver interview (and less frequently through direct observation or testing) in the clinic. These assessments are only performed periodically and typically rely on (caregiver) recall, hence representing a frequently biassed snapshot of the patient’s function captured in a clinical environment. Moreover, these conventional methodologies lack sensitivity to subtle symptoms in the early stages of the disease [3] and have limited utility as outcome measurements in clinical trials of interventions in preclinical AD. The development of current digital technologies provides an until now unavailable opportunity to repeat and improve measurements of functional decline in AD [4], which is exactly the aim of the “Remote Assessment of Disease and Relapse – Alzheimer’s Disease” (RADAR-AD) project [5].

Remote Monitoring Technologies (RMTs), such as fixed sensors at home, smartphone applications, and wearables (e.g. activity trackers), are minimally invasive and can measure parameters continuously and objectively. RMTs are suitable to monitor ADLs, since they are able to assess processes in a “real-world” environment, and therefore carry the promise of providing greater ecological validity and sensitivity than conventional in-clinic measures of function. The RADAR-AD study will use RMTs as the main method for assessing function via ADLs.

The field of digital technologies is rapidly developing. A recent review [4] emphasizes the increasing number of studies utilizing RMTs in both cognitively healthy older adult populations and patients with MCI or dementia. The authors conclude that only a few studies deal with real-world measurements. The number of studies using wearables is limited, and these studies generally cover only short-term measurement periods (< 4 weeks). Hence, it remains unclear which device outcomes have the most consistent association with cognitive and functional decline. Additionally, an important knowledge gap in the current literature is whether RMTs can detect the earliest symptoms of functional decline in preclinical AD patients.

The RADAR-AD study is a step forward to address some of these knowledge gaps. Its overall aim is to assess whether RMTs are able to accurately measure function with improved sensitivity in a real-world environment across preclinical-to-moderate stages of AD. In order to meet this aim, three sub-topics will be addressed:

1. Differentiation: is it possible to differentiate between healthy controls and participants with preclinical AD, prodromal AD, and mild-to-moderate AD using outcome measures of multiple RMTs?
2. Head-to-head comparison with current clinical standard: are the outcomes of the RMTs better than the standard clinical measures of functional decline?
3. Feasibility: what is the technical performance of RMTs in the real-world and how acceptable are RMTs to AD spectrum patients and their study partners?

## Methods

### Study design

RADAR-AD is a multicentre observational, cross-sectional, cohort study in subjects within the preclinical-to-moderate AD spectrum as well as healthy controls. The design entails three tiers: (1) main study, which includes smartphone applications and wearable devices only; (2) first sub-study, which in addition includes fixed sensors at the participant’s home; and (3) second sub-study, which in addition includes fixed sensors in an existing smart home environment.

Data will be collected in 13 countries across Europe (see Table 1). Participating clinical sites were selected based on their geographic location, expertise in digital technologies and disease population of interest, and the availability of clinical cohorts with known AD biomarkers.

**Table 1:** Participating clinical sites

Country	City	Memory clinic
France	Lille	Centre Hospitalier Régional Universitaire de Lille
Germany	Mannheim	Zentralinstitut für Seelische Gesundheit Mannheim
Greece	Thessaloniki	Aristotle University of Thessaloniki
Italy	Brescia	IRCCS Centro San Giovanni di Dio Fatebenefratelli
Norway	Stavanger	SESAM - Centre for Age-Related Medicine
Portugal	Lisbon	Faculdade de Medicina da Universidade de Lisboa
Romania	Bucharest	Carol Davila University of Medicine and Pharmachy
Slovenia	Ljubljana	Ljubljana University Medical Centre
Spain	Barcelona	Fundació ACE
Sweden	Stockholm	Karolinska Institutet
Switzerland	Geneva	Hôpitaux Universitaires de Genève
The Netherlands	Amsterdam	Amsterdam UMC
UK	Oxford	University of Oxford
	London	King’s College London

### Participants

The aim is to include 220 participants in the main study, of which a representative sub-sample of 40 participants continues into the first sub-study and 10 participants continue into the second sub-study. Each clinical site aims to recruit 5 participants of each study group (controls, preclinical AD, prodromal AD/MCI and mild-to-moderate AD; see below) in the main study, to equally distribute participants over the centres across Europe. Participants will be recruited from memory clinics and ongoing observational studies. Participants in the first sub-study are drawn from the four study groups, and are participants from the clinical sites in Amsterdam, Stavanger, Oxford, and London that already participated in the main study and meet the inclusion criteria for the sub-study. Participants in the second sub-study are participants from the clinical site in Thessaloniki only that already participated in the main study. To ensure all AD participants have underlying AD pathology, all participants will have AD biomarker evidence of supra-threshold Aβ burden (defined through each site’s local procedures), with either positron emission topography (PET) or cerebral spinal fluid (CSF), before inclusion. All healthy control participants will be cognitively unimpaired, be age- and sex-matched to the AD groups, and have confirmation of negative AD biomarkers if available. The diagnosis of subjects within the AD spectrum will be based on the NIA-AA criteria [2] and the study groups on the Mini-Mental State Examination (MMSE) [6] and the Clinical Dementia Rating (CDR) [7]:

1. AD spectrum (positive Aβ biomarkers)
  - Preclinical AD: MMSE > 26, CDR = 0 (*N* = 55)
  - Prodromal AD/MCI: MMSE > 23, CDR = 0.5 (*N* = 55)
  - Mild-to-moderate AD: MMSE > 17, CDR > 0.5 (*N* = 55)
2. Control (negative Aβ biomarkers if available)
  - Healthy controls: MMSE > 27, CDR = 0 (*N* = 55)

Inclusion criteria for all participants are male or female over 50 years of age, in generally good health, or diagnosed with mild chronic disorder (of metabolic, respiratory, immunological, cardiologic, and metabolic origin) or any other medical conditions that are controlled by therapy and/or do not impair function on a secondary basis to that of AD-related symptomatology, and availability of a study partner (spouse/family member/caregiver/friend) that consents to collaborating with the study. Furthermore, the participant and study partner should be able to read and communicate in the language of the recruitment centre and able to actively engage in tests and questionnaires, and the participant and study partner should both own a smartphone. For those volunteering in the first sub-study, availability of appropriate Wi-Fi and/or phone line connectivity in the participants’ house is required. For those volunteering in the second sub-study, participants and their study partners are required to move in to the smart home and should therefore only involve cognitively unimpaired age-matched participants. Exclusion criteria for all subjects are presence of a comorbid-neurological or psychiatric disease that may affect ADLs or social interactions, and any other kind of disorders that may significantly affect mobility, ADLs, or social interactions (e.g., immune-mediated inflammatory disorders, recovery from recent trauma, stroke, etc.). Since the local investigator has medical history available of potential participants for pre-screening, the local investigator is in the best position to judge the influence of these diseases or disorders on functional or social behaviour.

### Sample size

The web-based version of the Amsterdam Instrumental Activities of Daily Living (Amsterdam IADL) questionnaire [8] was selected as the primary endpoint for sample size calculation. This endpoint will serve as the gold standard for quantifying ADLs and staging the severity of functional impairment.

The hypothesis of the RADAR-AD study is that RMTs should have at least the same differentiating power as the Amsterdam IADL questionnaire in indexing functional decline in AD. Sample size was chosen to have 80% power to detect a difference in ADLs of > 10% between subjects in the following comparisons, as observed using available data from the Amsterdam IADL rating scale: (I) healthy controls vs. preclinical AD, (II) preclinical AD vs. MCI, and (III) MCI vs. mild-to-moderate AD. The lowest number of participants per group giving RADAR-AD’s cohorts at least 80% power is 55 in each of the four study groups, producing a total sample size of 220. This is based on the interquartile ranges from Jutten et al. [8] that reported on one thousand clinical trials to simulate sample size. They considered a trial positive if all three of the abovementioned comparisons had Holm adjusted *p*-values of less than 0.05. Each comparison was performed using a two-sided t-test with unequal variances.

The sub-studies are explorative studies, which primary focus is on feasibility of the devices. To meet this goal, a small subset of participants from the main study is sufficient for the sub-studies.

### Patient advisory board

A patient advisory board (PAB) was set up in month 3 of the project. This work has been led by Alzheimer Europe in collaboration with project partners. The PAB has provided feedback to the study protocol, patient-facing materials, prioritization of functional domains, selection of devices, and ethical issues. Further information about the PAB composition and work can be found at [9].

### Selection of relevant functional domains

We carried out an extensive literature review to select functional domains relevant to AD biomarkers, quality of life (QoL), rate of disease progression, and loss of independence. PubMed literature until March 2019 was searched using relevant keywords and the results were tabulated to guide the prioritization of these preliminary functional domains. A more detailed description of the prioritization of the functional domains process can be found in [10].

Functional domains were ranked and grouped by the empirical evidence for each in relation to ability to predict MCI-to-AD dementia conversion, relevance to early AD, and prediction of decline in people with dementia. The list of functional domains from the literature review then underwent a systemic Delphi-type process before being handed over for discussion with the RADAR-AD Patient Advisory Board for feedback on the relevance of the functional domains to the experience of living with AD or caring for someone with AD from patients and/or their carers. The results from these search criteria were prioritized into tiers: tier 1, highly relevant; tier 2, relevant; tier 3, neutral; and tier 4, less relevant. Functional domains that met all three established criteria (predicts MCI-to-AD conversion, relevance to early AD, and being predictive of decline in people with dementia) and were reported as relevant by the RADAR-AD Patient Advisory Board were grouped into tier 1. Functional domains that met two of the criteria and were reported as relevant by the RADAR-AD Patient Advisory Board were grouped into tier 2. Functional domains that met one of these criteria and were reported as relevant by the RADAR-AD Patient Advisory Board were grouped into tier 3, and functional domains that met 1 of the criteria were grouped into tier 4. The result can be found in the first column of Table 2.

**Table 2:** Functional domains and their tests for both study participant and study partner

Functional domain	RMT	Paper pencil test
1. Difficulties at work	AIADL (via email)^1^	AIADL (in clinic)^2^
2. Spatial navigation and memory	Altoida MD^1,2^, pRMT^1^, AIADL (via email)^1^	ECog^2^, AIADL (in clinic)^2^, ADCS-ADL^2^
3. Planning skills and memory required for task-completion	Altoida MD^1,2^, Mezurio^1^, AIADL (via email)^1^	DSST^2^, E-Cog^2^, AIADL (in clinic)^2^, ADCS-ADL^2^, CERAD^2^
4. Managing finances	Banking app^2^, AIADL (via email)^1^	ECog^2^, AIADL (in clinic)^2^, ADCS-ADL^2^, SFS^2^
5. Self-care	AIADL (via email)^1^, RAS^1^, Camera^1^	AIADL (in clinic)^2^, ADCS-ADL^2^, SFS^2^
6. Self-management, e.g., running errands and shopping	Mezurio^1^, AIADL (via email)^1^, RAS^1^	E-Cog^2^, AIADL^2^, ADCS-ADL^2^, SFS^2^, NPI^2^
7. Acquiring new skills	Mezurio^1^	CERAD^2^
8. Sleep quality and circadian rhythms	Fitbit^1^, Axivity^1^, Mezurio^1^, DREEM^1^	PSQI^2^, ESS^2^, NPI^2^
9. Use of technology/devices	Altoida MD^1,2^, pRMT^1^, Mezurio^1^, AIADL (via email)^1^	ECog^2^, AIADL^2^, SFS^2^, Smartphone proficiency test^2^
10. Dysnomia, word-finding difficulties	Mezurio^1^	CERAD^2^
11. Gait	Fitbit^1^, Axivity^1^, Gait Up^2^	E-Cog^2^, ADCS-ADL^2^
12. Difficulties driving	CANEdge^1^, AIADL (via email)^1^	E-Cog^2^, AIADL (in clinic)^2^
13. Interpersonal interaction	pRMT^1^, Camera^1^	SFS^2^
14. Motivation, signs of apathy or withdrawal	pRMT^1^, Mezurio^1^, Camera^1^	SFS^2^, NPI^2^

^1^Used in participant’s own environments

^2^In-clinic test

*ADCS-ADL* Alzheimer’s Disease Cooperative Study—Activities of Daily Living scale; *AIADL* Amsterdam Instrumented Activities of Daily Living; *Altoida MD* Altoida Medical Device; *Axivity* Axivity AX3 activity tracker; *Camera* Autographer wearable camera; *CANEdge* CANedge driving performance logger; *CERAD* CERAD neuropsychological test battery, consisting of verbal fluency (animal naming), 15-item Boston Naming Test, word list learning, word list recall, word list recognition, Rey Complex Figure drawing and recall; *DREEM* DREEM device; *DSST* Digit Symbol Substitution Test; *Ecog* Everyday cognition; *ESS* Epworth Sleepiness Scale; *Fitbit* Fitbit Charge 3 activity tracker; *Gait Up* Gait Up Physilog sensors; *Mezurio* Mezurio smartphone application; *NPI* Neuropsychiatric Inventory; *pRMT* RADAR passive RMT app; *PSQI* Pittsburgh Sleep Quality Inventory; *RAS* residential activity sensors; *SFS* Social Functioning Scale

### Device selection

Based on the list of relevant functional domains, a device selection process of potential RMTs was initiated to quantify behavioural, motor and cognitive measures in each functional domain. A selection process was developed that would bring together technical, clinical, and participant perspectives, based on three interlinked activities to *identify* relevant technologies, *synthesize* into a compatible selection, and *verify* that the rationale for that selection is well-founded [10]. Devices were selected with the following considerations in mind:

1. *Features*. Size, battery life, comfort, convenience, or other relevant features such as whether it is water-resistant.
2. *What the RMT can do for the participant*. How the RMT is perceived by the participant and his/her caregiver, if it provides feedback regarding exercise or steps per day or shows time or date, and if it does not compound potential societal stigmas. These issues were discussed extensively with the PAB.
3. *What the RMT can do for the researchers*. Provide information about relevant processes, type of data provided, presence of CE marking as medical device, and assess remotely.

**Fig. 1:**
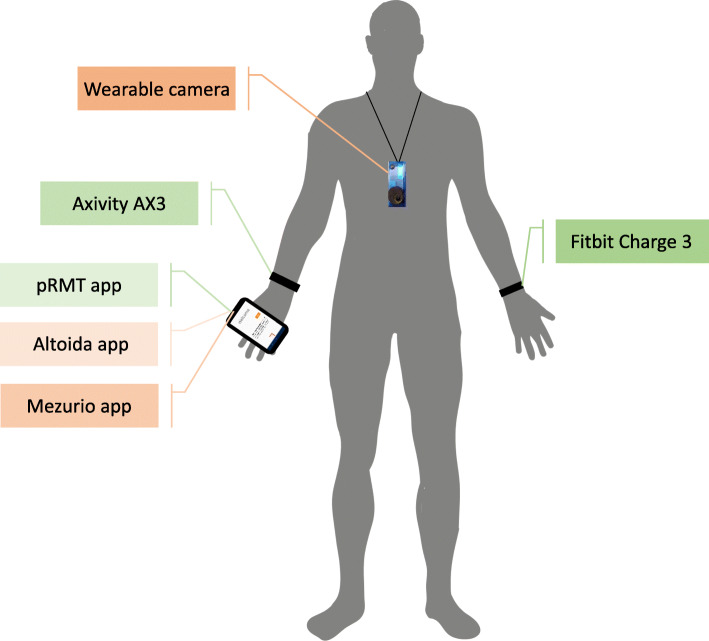
Representation of the smartphone applications and wearable devices (main study) on a right-handed model participant. Devices in green measure continuously while devices in orange measure periodically

Selected devices were discussed with the RADAR-AD PAB in order to ensure that patients and their study partners are willing and able to use the RMTs. The following RMTs were chosen for use in the RADAR-AD main study in the participant’s own environments (Fig. 1):

1. *Fitbit Charge 3*. A wrist-worn accelerometry device that will be worn on the non-dominant arm, which will provide information about night-time sleep duration and number of awakenings, heart rate, and daytime activity profile. This CE-marked device was chosen because it measures both heart rate and activity. Moreover, the RADAR-AD PAB indicated that they appreciate an aesthetic device, which gives feedback on their activities.
2. *Axivity AX3*. An accelerometry device which will be worn on the dominant hand. It passively measures raw 3D acceleration, which will be further processed to obtain measures of sleep, physical activity, and circadian rhythms. This CE-marked device was added to the Fitbit Charge 3 since it provides a more granular measure of raw accelerations, unlike the Fitbit, but the AX3 has no display to provide feedback to the user and does not measure heart rate. The large UK Biobank study (*N* = 100,000) has already demonstrated AX3’s suitability to measure activity profiles, while also providing data from a cognitively normal cohort [11].
3. *RADAR-base passive RMT app* (*pRMT*). A smartphone application for Android users only that will passively collect information from app usage, relative GPS (Global Positioning System) coordinates, Bluetooth, and other on-phone sensors [12] for further analysis of mobility and social communications [13]. This app does not need any active involvement of the participant, while collecting large amounts of data on social behaviour and phone usage.
4. *Autographer wearable camera*. This device will be worn around the neck and will be taking a photo every 20 s in order to contextualize the activities measured using the Fitbit and Axivity wristbands. This camera is CE marked, is lightweight, has a wide-angle lens, and captures images unobtrusively of the participant’s perspective. Wearable cameras are already used in studies of social interactions, sedentary, behaviour and health-related research [14]. The use of the camera is optional for participants, since participants might have privacy concerns [15].
5. *Mezurio app*. A smartphone application for iOS and Android based on the very earliest neural and cognitive correlates of preclinical AD, utilizing both active and passive data collection. Participants will be asked to interact with the app for no more than 10 min at a regular time of their own choosing, during each day of the study. A bespoke protocol for the app containing a mix of different gamified tasks and short self-report questionnaire measures has been designed for the RADAR-AD study. Mezurio also includes active tasks for voice and speech analysis and passive measurement of typing dynamics, and GPS will be collected for iOS users only. The caregiver will also be asked to complete a complementary parallel Mezurio schedule of self-reported measures relating to the participant’s capabilities across functional domains. A previous study shows the feasibility of this app [16] and a large ongoing study (*N* > 16,000) will provide normative data [17].
6. *Altoida Medical Device* (*Altoida MD*). A smartphone and tablet-based CE-marked digital biomarker platform that simulates a complex ADL exercise using augmented reality. It calculates the Neuro Motor Index (NMI), a score derived from the performance combining data streams from voice data, hand micro movements and errors, gait micro errors, posture changes, eye tracking, and visuospatial navigation micro errors. It has been shown to have an accuracy of 94% in predicting cognitive worsening in amyloid positive individuals who converted from MCI to AD after 5 years [18]. The Altoida MD app will be used in the clinic baseline and weekly at home.
7. *Amsterdam IADL*. A questionnaire, filled out by a caregiver, assessing instrumented ADLs (IADLs) on a PC or tablet. The questionnaire is validated in a memory clinic cohort [19] and is able to detect functional decline [8]. The Amsterdam IADL will both be filled out in the clinic baseline visit and weekly at home.

The following RMTs were chosen to use in the RADAR-AD main study in the clinic:

1. *Gait Up Physilog sensors*. These body-worn sensors contain accelerometers and gyroscopes to calculate 26 gait parameters, such as walking speed, gait asymmetry, and step variability during a 1-min walking task, a 1-min dual task (in which people have to count back from 100 to 0 while walking), and a timed up-and-go (TUG) test. This CE-marked RMT was chosen because it is user-friendly and validated in a large number of patient groups and normative data for cognitively healthy older adults are available [20].
2. *Banking app*. A smartphone application simulating a bank withdrawal. The application was developed as part of a multi-sensor assessment and monitoring system, during the Dem@Care project [21], to assess parameters related to the functional abilities of managing finances. The app is now extended and enhanced for use in this study (e.g., multi-lingual use).

The following fixed RMTs were chosen to use in the first RADAR-AD sub-study:

1. *Residential activity sensors*. Presence sensors (based on infrared motion), door-window sensors (based on magnetic sensors), and appliance energy-usage sensors (measuring energy consumption) will be strategically placed in the participants’ homes. These sensors will monitor occupancy of certain spaces (e.g., the kitchen) and utility usage (e.g., opening the fridge door or kitchen appliances usage). Fusing those values together via complex event processing methods will then provide profiling, context-awareness, and activity recognition related to IADLs, such as preparing a meal. Outlier detection can detect certain problems and symptoms, for example long duration of chores or visiting the bathroom multiple times at night.
2. *DREEM headband*. A handband-based remote polysomnography device, worn during sleep that logs parameters on sleep quality and patterns through dry electroencephalogram (EEG) electrodes and accelerometers [22]. This data can, among others, be used to assess the distribution of the various sleep stages.
3. *CANedge driving performance logger*. A data logger that is plugged into the Open DataBase Connectivity (ODBC) port of the participant’s car (if available). This logger, which is generally used to perform car diagnostics, gathers data on acceleration and breaking patterns, driving frequency, and fuel consumption. The advantage of this device over, e.g., a real-world driving evaluation is that it measures driving behaviour each time a participant uses the car for 4 weeks, it does not affect someone’s driving behaviour, and it does not need an additional contact moment.

For the second sub-study, 10 healthy control participants from the main study will be observed for 1 week in the Digital Home developed by the Centre for Research and Technology Hellas (CERTH). The CERTH-ITI nZEB Smart House [23] is a rapid prototyping and novel technologies demonstration infrastructure resembling a real domestic building where occupants can experience actual living scenarios while exploring various innovative smart Internet of Things (IoT)-based technologies with provided energy, health, big data, robotics, and artificial intelligence (AI) services. Technology related to dementia assessment and monitoring for elders has been embedded as part of H2020 ACTIVAGE [24] and FP7 Dem@Care [25] and will be enriched with first sub-study RADAR-AD devices but in greater variety and quantity so as to explore the potential of deeper and wider monitoring capabilities to extract biomarkers in a controlled environment.

### Data platform

Selected devices were then integrated by a development team into the RADAR-base platform, an open-source mHealth platform developed by the RADAR-CNS consortium [13]. Based on the Apache Kafka stream processing engine, it harmonizes the data it receives using a set of standard schema and typically stores its data in Apache Avro file format. Clinical research forms (CRFs) were built using the REDCap system [26, 27]. The complete platform was then deployed in a secure, private, OpenStack cloud environment physically within the Oxford University Big Data Institute’s High Performance Biomedical Compute facility.

### Study timeline and procedures

Eligible participants and their study partners will receive written and oral information regarding the study. There will be a minimum time window before the RADAR-AD research team will contact the potential participant and study partner again to answer any questions and to determine if they would like to participate in the study. If both are willing to participate, the baseline and close-out visit will be scheduled, with at least 8 weeks between the two appointments. Informed consent will be signed before participation in the study.

During the baseline visit, several paper and pencil tests will be administered (see Table 2). In order to compare the outcomes of the RMTs with the outcomes of standard clinical tests and to confirm clinical diagnosis, participants will complete a battery of neuropsychological tests, conventional paper and pencil assessments, and a physical examination in a clinical setting. Furthermore, all smartphone applications will be installed on the participant’s and their partner’s phones, and instructions will be provided on how to operate the RMTs at home. Both the participant and the study partner have to be present at the baseline visit, which will take around 4 h.

**Fig. 2:**
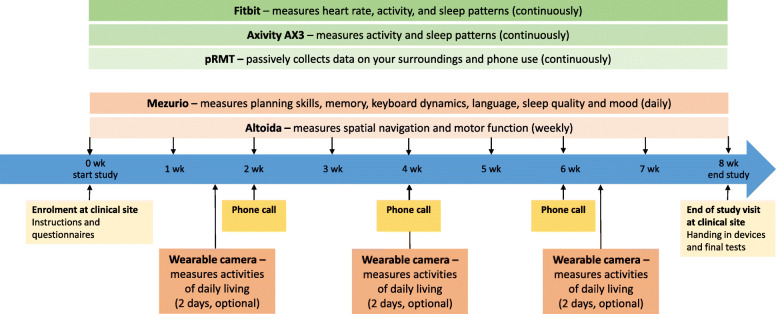
Timeline of the visit scheme of the main study. Participants visit the clinic for a baseline visit. Both standard clinical tests and digital tests will take place during this baseline visit. Additionally, participants will receive a description and training for the device usage at home. The participants will use the RMTs for 8 weeks at home. Devices in green measure continuously while devices in orange measure periodically. The participants will be called by phone every 2 weeks to evaluate the device usage and to check for adverse events. After these 8 weeks, the participants will visit the clinic again for a close-out visit, in which the devices have to be handed in and several final tests will be done

**Fig. 3:**
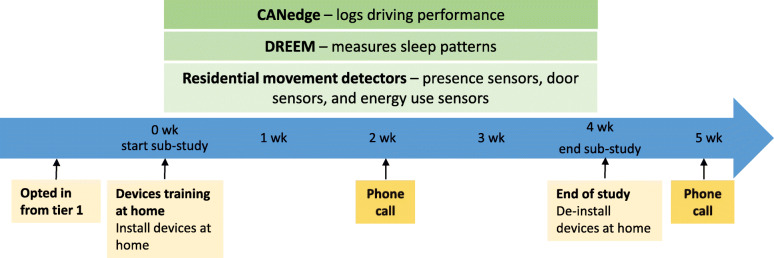
Timeline of the first sub-study. Participants will be opted in as a representative sub-sample from the main study equally distributed over the study groups. Several residential activity sensors will be installed in the home of the participant and the participant will receive training regarding the devices. During the 4-week data collection, participants will use the DREEM device each night. After 2 weeks, a phone call will be made to evaluate the device usage and to check for adverse events. After 4 weeks, the devices will be de-installed at home

Participation in the main study lasts for 8 weeks, with an optional extension of 4 weeks for the first sub-study (for participants in Amsterdam, Stavanger, Oxford, and London only) or 1 week for the second sub-study (for participants in Thessaloniki only). The first sub-study can also be done in parallel to the main study. The study visit scheme can be found in Fig. 2 (main study) and Fig. 3 (first sub-study). The banking app, Altoida MD and walk tests will be performed during the baseline visit in clinic. The Fitbit, Axivity (sampled at 25 Hz), and pRMT app will measure passively and continuously during the 8-week period of data collection. The optional wearable camera will be worn during 2 consecutive days on up to three occasions. The Mezurio schedule has been set-up for active involvement on a daily basis, while the Altoida MD and Amsterdam IADL Questionnaire need active involvement on a weekly basis. Every 2 weeks, a phone call is made by a RADAR-AD researcher to participants to check for technical issues and adverse events. Additionally, compliance is checked and discussed with the participant. The only RMTs used by the study partner are the Amsterdam IADL and Mezurio app in order to answer questions about the participant on a weekly and daily basis, respectively.

### Feature extraction

High-frequency sensor data are typically processed to extract meaningful features using algorithms which are highly specific to that stream and then aggregated over a variety of time periods. For example, an accelerometer providing 30 measurements per second can be processed [28] to derive sleep characteristics for each night, and relative location data can be used to estimate distance travelled per day, duration of home stay per day, number of places visit per day, etc. Work is now ongoing to curate relevant feature algorithms into a computationally consistent environment so that a database suitable for further analysis can be produced.

### Outcomes

The primary outcome of this study is the difference in functional domain profiles using each RMT separately between the four study groups: healthy controls, participants with preclinical AD, participants with prodromal AD, and participants with mild-to-moderate AD. A secondary outcome is the association between the RMTs and the standard clinical tests assessing the same functional domain. The additional objective of the second sub-study is to set up and validate the capabilities of a fully equipped, digital home of the future, with an extended set of monitoring equipment and analysis from the first sub-study.

### Statistical analysis plan

Data obtained from RMTs will be collected continuously (for example, accelerometer recordings or GPS data), periodically (for example, the weekly Altoida or Amsterdam IADL), or obtained during the baseline visit only (for example, gait assessment). Continuous data will be subjected to feature extraction as per the “Feature extraction” section. We will then analyse each RMT dataset separately to assess whether they differ significantly between the four study groups, and whether they correlate with standard clinical questionnaires.

For a continuous day-by-day time series, we will model such a RMT measure using a mixed-effect model for repeated measures (MMRM) based on observed case data. The effect size of any between study groups differences (or relation to related ADL scale) is of primary interest. In the case of RMT data obtained at a single study time point (such as gait assessment), analysis of covariance (ANCOVA) will be conducted. Correction for multiple comparison will be considered for univariate RMT modelling.

As a second step, we will attempt to answer the question, whether a combination of different RMT assessments contributes to either study group discrimination and/or is related to ADL scale in a multivariate predictive modelling approach. Results of univariate modelling will be considered a filter-based feature selection, which will inform what information to include into the model. Continuous RMT data will be aggregated (e.g. mean, standard deviation, lagged autocorrelation, as estimated using data from a whole 8-week experiment duration) for each participant. These preselected aggregated features in combination with preselected RMT measures obtained at a single study point will be used for modelling. Since many classification or regression algorithms cannot handle situations where a value of at least one covariate is missing for a particular data sample, imputation will be applied, instead of completely removing this data sample. This imputation will be done under assumption that data are missing at random, and where the number of missing cases is not drastically high, e.g. less than 25%. Multivariate prediction (of either study group or related ADL scale) models will be constructed and further variable selection, based on, for example, LASSO L1-regularization for (generalized) linear models, or different feature selection algorithms will be applied. Models’ performance will be characterized through *n*-fold cross-validation.

For the assessments which will be primary done in the clinic and repeated at home (as, for example, experiments with the Altoida app), results will be compared using Bland Altman analysis to assess the agreement between two quantitative variables. Based on the result, recommendation regarding possibility to use home-based tests will be provided. To check whether “real-world” (at home) assessment outperforms the one done in the “clinical environment”, we will check whether the predictive models (described above) using “real-world” assessments have better predictive performance than the ones using “clinical” assessments.

### Modelling of the data

To allow modelling-driven interpretation of the cross-sectional data that will be collected during the main study and two sub-studies, we will also model functional and cognitive decline using data from available longitudinal cohorts such as Alzheimer’s Disease Neuroimaging Initiative (ADNI) [29] and AddNeuroMed [30], which will provide variables of interest. After generation of these overview variables, initial steps for statistical and machine learning based modelling will be performed. These initial steps for modelling will include extraction of individual item scores of variables, pre-processing, and normalizing the data. We will then apply and compare statistical (linear mixed models) and machine learning based approaches (e.g., recurrent neural networks) to model the functional and cognitive measures obtained from the heterogeneous longitudinal cohorts over time, having identified biosignatures indicative of changes in functional and cognitive status.

In addition, we have established semantic mappings between digital biomarkers and functional domains. A further aim is to understand, whether the digital biomarker data collected in RADAR-AD can be mapped to the longitudinal model.

Our work also aims to build and evaluate different machine learning classifiers, for example random forest and gradient boosted decision trees trained on functional domains. These classifiers will be built on multiple longitudinal datasets, such as ADNI and National Alzheimer’s Coordinating Center (NACC). It will help us to analyse the difference of functional domains between the different AD stages. The prioritization of functional domains will be mapped with those established from literature [10]. This classifier will be further applied on the digital data in order to see if RTMs are a more sensitive measure of disease progression compared to the traditionally clinically measured functional and cognitive data.

### Ethics

Medical ethics committees associated with each clinical site will approve the study independently. Minor adjustments of the research protocol, such as a substitution of a clinical questionnaire, are allowed in each country in order to facilitate ethical approval. Before submission to the local ethical committees, the study was reviewed by our internal ethics group (Lucivero et al., in preparation). The study will be carried out in accordance with the ethical conduct and juridical laws of the Declaration of Helsinki of 2013. Participants will be recruited from memory clinics and ongoing cohort studies. Appropriately trained research staff at the clinical sites will screen participants for eligibility. All study participants and their study partners will sign written informed consent separately before inclusion in the study. In all cases, participants’ data will be collected and processed according to the European General Data Protection Regulation (GDPR) and any applicable national privacy regulation. The RMTs data storage and utilization will comply with EU regulation No. 1291/2013 of the European Parliament and of the Council of 11 December 2013 and the new Medical Device Regulation (EU 2017/745).

### Privacy and data processing

Participant’s privacy and confidentiality will be respected throughout the course of the study. All clinical and personal data (age, gender, level of education, clinical data, and diagnosis) will be provided with a code that cannot be traced to an individual. Identifiable information collected at clinical sites will be stored within a password protected key file, separate from the RADAR-base platform and accessible only to members of the local research team. All RMT data will be centrally stored in the RADAR-base platform.

Data collected via the Fitbit Charge 3 and Altoida MD is first transmitted to respective company data warehouses from which data will be accessed, encrypted, and uploaded to a secure server maintained by the sponsor organization and will be not identifiable by participant name. Data collected via smartphone will be encrypted and uploaded to the secure RADAR-base server by Wi-Fi or mobile data connection. Data will be temporarily cached on the smartphone until an appropriate connection is available and will then be automatically deleted from the phone’s memory.

The non-identifiable data acquired may be transmitted through a computer network, through the internet, or transferred via removable media to be shared with other members of the RADAR-AD consortium. This information will be anonymized and will not include anything that could identify participants by name, date of birth or address. Participants will be informed and asked to consent to sharing of information.

The RADAR-AD research team will keep legible and accurate documents to ensure thorough documentation of study conduct. The highest degree of confidentiality will be maintained for managing data collected throughout the course of this study, however, to meet legal responsibilities and quality assurance policies, the investigational site will permit authorized representatives of the sponsor, funder, and health authorities to examine anonymized records to satisfy quality assurance reviews, audits, and evaluations of study safety and progress.

## Results

The first participant was enrolled in July 2020, and recruitment will continue until December 2021. The first results are expected to be disseminated in 2022.

## Discussion

The RADAR-AD project aims to study the use of RMTs to measure function in participants across the AD spectrum. RMTs are potentially beneficial in comparison to standard clinical assessments because they can measure continuously during sleep, gait, and ADLs at home; do not rely on caregiver recall; are objective; and are minimally invasive and burdensome for the participant. Previous literature shows that measuring ADLs in the real-world is feasible using wearables and home-based sensors in dementia patients [4]. Healthy control studies with a large sample size have been carried out [11], but real-world data from large studies with AD spectrum patients are still lacking. Therefore, this study will contribute to the field significantly, since this is a relatively large cohort study using smartphone applications, wearables, and home-based sensors. Moreover, our cohort will complete an extensive battery of neuropsychological tests and conventional paper and pencil assessments of cognition and function. Assessments of cognition and function will be carried out both in the clinic and in the real-world and therefore allow direct comparison. Of great importance is that, in contrast to many previous studies, all clinical participants will have AD biomarker confirmation. This is in line with the recent NIA-AA research framework [2] and current clinical trials, which emphasize the importance of the outcomes of this study in defining endpoints for future clinical trials.

One of the main tasks of RADAR-AD is securing data privacy. To ensure data privacy, thorough precautions, as mentioned in the “Privacy and data processing” section, will be undertaken, including the use of the specifically designed data platform RADAR-base. A possible challenge is that older adults may distrust or not accept the technologies to observe them. However, despite this perception, the review of Piau et al. [4] and our RADAR-AD PAB workshop (Stavropoulos et al., in preparation) suggest that the acceptability issues are relatively minor, both with fixed sensors and wearables. Moreover, one of the aims of this study is to test the acceptability of RMTs to measure functioning in daily life, and therefore, any major acceptability issues will constitute an important outcome of the study. Another challenge is the large number of participating sites, i.e., fourteen, across thirteen countries. To ensure high data quality across all centres, several precautions are taken: a detailed study manual describing all study procedures, extensive training for all researchers involved before inclusion of the first participant in each centre, and data quality will be monitored centrally during the data collection period. It is expected that AD patients have a less structured day and are less active during the day compared to unimpaired older adults [31–34]. Also, cultural differences between countries are expected, since, e.g., older adults in the Mediterranean countries make less use of computers and smartphones than older adults in Northern European countries [35].

### Limitations

One limitation is the difficulty to obtain independent behavioural data to validate the RMT metrics. We emphasize that the RADAR-AD study is an exploratory study aiming to find differences between the four study groups on the RMT outcomes and the association with standard clinical tests, but this does not necessarily validate the real-world performance of the RMTs. Longitudinal assessment of functional decline and the associations with RMTs aiming to measure this decline will help with validation. However, the design of the RADAR-AD study is cross-sectional. Therefore, if the results of this study are promising, longitudinal studies are recommended for further research. Another limitation is the start of the study in the middle of the COVID-19 pandemic. Behaviour of individuals may have changed, especially for elderly people at high risk. Therefore, COVID-19 restrictions across countries will be observed closely. Moreover, medical events such as COVID-19 positive tests will be monitored during the study by asking the participants about medical events during the bi-weekly phone calls and end-of-study visit. Most importantly, this pandemic demonstrates the importance of monitoring disease symptoms remotely, and therefore we are confident that the RADAR-AD study is of added value.

## Conclusions

The RADAR-AD study aims to assess the accuracy and validity of RMTs in measuring functional decline in a real-world environment across preclinical-to-moderate stages of AD compared to standard clinical rating scales. If successful, the RADAR-AD study may provide important guidance on novel digital endpoint deployment for future clinical trials. Improved measures and monitoring of function may ultimately lead to higher successful drug development rate, earlier interventions, and overall better care of those affected by AD.

## Data Availability

Not applicable.

## Abbreviations

AD: Alzheimer’s disease
ADCS-ADL: Alzheimer’s Disease Cooperative Study—Activities of Daily Living scale
ADL: Activities of daily living
ADNI: Alzheimer’s Disease Neuroimaging Initiative
AI: Artificial intelligence
Altoida MD: Altoida Medical Device
Amsterdam IADL/AIADL: Amsterdam Instrumental Activities of Daily Living Questionnaire
ANCOVA: Analysis of covariance
Aβ: Amyloid β
CDR: Clinical Dementia Rating
CE: Conformité Européenne
CERTH: Centre for Research and Technology Hellas
CRF: Clinical research form
CSF: Cerebral spinal fluid
DSST: Digit Symbol Substitution Test
Ecog: Everyday cognition
EEG: Electroencephalogram
ESS: Epworth Sleepiness Scale
GDPR: General Data Protection Regulation
GPS: Global Positioning System
IADL: Instrumented Activities of Daily Living
IoT: Internet of Things
MCI: Mild cognitive impairment
MMRM: Mixed-effect model for repeated measures
MMSE: Mini-Mental State Examination
NACC: National Alzheimer’s Coordinating Center
NIA-AA: National Institute on Aging—Alzheimer’s Association
NMI: Neuro Motor Index
NPI: Neuropsychiatric Inventory
ODBC: Open DataBase Connectivity
PAB: Patient advisory board
PET: Positron emission topography
pRMT: RADAR-base passive RMT app
PSQI: Pittsburgh Sleep Quality Inventory
QoL: Quality of life
RADAR-AD: Remote Assessment of Disease and Relapse—Alzheimer’s Disease
RAS: Residential activity sensors
RMT: Remote monitoring technology
SFS: Social Functioning Scale
TUG: Timed up-and-go
